# Natural Selection of H5N1 Avian Influenza A Viruses with Increased PA-X and NS1 Shutoff Activity

**DOI:** 10.3390/v13091760

**Published:** 2021-09-03

**Authors:** Aitor Nogales, Laura Villamayor, Sergio Utrilla-Trigo, Javier Ortego, Luis Martinez-Sobrido, Marta L. DeDiego

**Affiliations:** 1 Center for Animal Health Research, CISA-INIA-CSIC, Valdeolmos, 28130 Madrid, Spain; sergio.utrilla@inia.es (S.U.-T.); ortego@inia.es (J.O.); 2 Department of Molecular and Cell Biology, Centro Nacional de Biotecnología (CNB-CSIC), Campus Universidad Autónoma de Madrid, 28049 Madrid, Spain; lvillamayor@cnb.csic.es; 3 Department of Disease Intervention and Prevention, Texas Biomedical Research Institute, San Antonio, TX 78227, USA; LMartinez@txbiomed.org

**Keywords:** influenza A virus, NS1, PA-X, HPAIV, H5N1, shutoff

## Abstract

Influenza A viruses (IAV) can infect a broad range of mammalian and avian species. However, the host innate immune system provides defenses that restrict IAV replication and infection. Likewise, IAV have evolved to develop efficient mechanisms to counteract host antiviral responses to efficiently replicate in their hosts. The IAV PA-X and NS1 non-structural proteins are key virulence factors that modulate innate immune responses and virus pathogenicity during infection. To study the determinants of IAV pathogenicity and their functional co-evolution, we evaluated amino acid differences in the PA-X and NS1 proteins of early (1996–1997) and more recent (since 2016) H5N1 IAV. H5N1 IAV have zoonotic and pandemic potential and represent an important challenge both in poultry farming and human health. The results indicate that amino acid changes occurred over time, affecting the ability of these two non-structural H5N1 IAV proteins to inhibit gene expression and affecting virus pathogenicity. These results highlight the importance to monitor the evolution of these two virulence factors of IAV, which could result in enhanced viral replication and virulence.

## 1. Introduction

Influenza A viruses (IAV) belong to the *Orthomyxoviridae* family of viruses containing an eight-segmented, single-stranded, negative-sense RNA genome. IAV are divided into subtypes based on the two glycoproteins on the surface of the virus: hemagglutinin (HA) and neuraminidase (NA). To date, 18 different HA subtypes and 11 different NA subtypes (H1 through H18 and N1 through N11, respectively) have been described [1,2]. Although IAV can infect and replicate in multiple mammalian and avian species, the natural host reservoir species are wild aquatic birds [2,3]. Avian influenza virus (AIV) is a type of IAV that is isolated from and adapted to avian host species, and it is classified as low or highly pathogenic (LPAIV and HPAIV, respectively) based on its pathogenicity in domestic chickens. LPAIV strains are associated with mild clinical signs in broilers and reduction in egg production in layers, but promote secondary infections causing an increase in mortality [4,5]. On the other hand, HPAIV strains are responsible for systemic and fatal infections with high mortality rates in poultry [4,5]. HPAIV H5N1 occurs mainly in birds, is highly contagious, and is deadly. The first HPAIV H5N1 was detected in 1996 in geese in China [6] and continues to spread, posing a major challenge to both animal and human health. In 1997, the first human case of an H5N1 HPAIV was reported in a three-year-old boy in Hong Kong, China [7]. The same year, a total of 18 human infections with H5N1 HPAIV were reported in Hong Kong, leading to 6 deaths [7]. These human cases were preceded by outbreaks in poultry, and it is very likely that human infections were acquired directly from infected birds, without the involvement of an intermediate host. The outbreak in Hong Kong was efficiently controlled by the slaughter of all poultry in markets and farms in 1997 [8]. However, H5N1 HPAIV continues to circulate and evolve among poultry in Asia, and zoonotic spillover was again observed in early 2003 [9]. Since January 2003 until now, the cumulative number of laboratory-confirmed human cases of H5N1 HPAIV infections were 861, including 455 deaths, in 17 different countries (Available online: https://www.who.int/influenza/human_animal_interface/2020_01_20_tableH5N1.pdf?ua=1 (accessed on 5 July 2021)), most of these human infections being due to the close contact with infected birds. People infected with H5N1 HPAIV developed severe respiratory disease, with symptoms including fever, cough, shortness of breath, and pneumonia, which frequently progressed to acute respiratory distress syndrome (ARDS) and multiorgan failure [10]. Systemic spread and cytokine storm [11] have been described as possible disease-aggravating factors of HPAIV H5N1 infections, but the reasons for their high virulence in humans remains unclear. Although the effective transmission of H5N1 HPAIV from humans to humans has not yet been observed, humans are at risk of an H5N1 HPAIV pandemic.

IAV non-structural 1 (NS1) protein is the main transcript of the NS segment and a key virulence factor, playing an important role in counteracting innate immune responses induced by the host through different mechanisms [12,13]. IAV NS1 inhibits activation of innate antiviral responses by sequestering double stranded (ds) RNA, a potential trigger of type I and III interferon (IFN) responses [14]. IAV NS1 also inhibits activation of IFN signaling through retinoic acid-inducible gene I (RIG-I), an intracellular sensor of virus infection, by binding to RIG-I and preventing its activation [15,16,17]. Moreover, the NS1 protein of some IAV strains interacts with components of the cellular pre-mRNA processing machinery, including the cleavage and polyadenylation specificity factor 30 (CPSF30), and poly(A)-binding protein II, inhibiting proper 3′ end processing and blocking the nuclear export of cellular mRNAs, which lead to the inhibition of host gene expression, including IFN and pro-inflammatory responses [18,19]. Some publications have shown the relevance of amino acid residues in IAV NS1 functions and virus pathogenesis. For example, a five amino acid deletion at position 80–84 described in H5N1 IAV isolated from 2000 to 2015 [20] has been implicated in enhancing virulence in ducks, chickens, and mice [21,22], at least in part by enhancing resistance to tumor necrosis factor (TNF)-induced responses [23]. Likewise, IAV NS1 mutations L103F and I106M in H5N1 IAV circulating since 1998 are responsible for inhibition of host gene expression by enhancing the interaction of H5N1 IAV NS1 with CPSF30 [24]. Moreover, these mutations have been shown to enhance viral virulence in mice by increasing the systemic spread of the virus from the lungs, mainly to the brain [25] and by mediating interstitial pneumonia in mice [26].

IAV segment 3 encodes the polymerase acidic (PA) and PA-X proteins [27]. PA-X is translated as a +1 frameshift open reading frame (ORF) from the PA viral segment [27]. During translation, the ribosome shifts at a specific sequence in the PA mRNA, a U-rich region followed by a rare codon, which usually promotes ribosomal shifting because they are typically decoded more slowly [28]. This ribosomal frameshifting results in the expression of PA-X, which shares the same first N-terminal 191 amino acids with PA, including the endonuclease domain, and encodes a unique, short C-terminal sequence [27]. Most of the human and avian IAV strains contain a 61 amino acid long C-terminal extension (leading to a 252 amino acid PA-X) [29]. By contrast, some IAV, including the 2009 human pandemic H1N1 (pH1N1), canine, and certain swine IAV, possess a TGG (W) to TAG (stop) mutation at codon 42 in the X ORF, leading to a 232 amino acid long PA-X protein [30]. IAV PA-X selectively degrades RNAs transcribed by host RNA polymerase II (Pol II) [31], contributing to host-cell shutoff and inhibition of host antiviral responses [27]. Moreover, PA-X modulates host inflammation, immune response, apoptosis, cell differentiation, and tissue remodeling [27]. Interestingly, the role of PA-X in IAV pathogenesis seems to be strain specific [12]. Loss of PA-X expression increased viral replication, pathogenicity, and host inflammatory response for pH1N1 in mice [32,33] and for H5N1 IAV in mice, chickens, and ducks [34]. In addition, PA mRNA and protein synthesis were upregulated in PA-X-deficient pH1N1 and H5N1 virus-infected cells [33]. Using viral strains of pH1N1, H5N1 HPAIV, and a H9N2 LPAIV, it has been shown that viruses with full-length PA-X (252 amino acids) replicate more efficiently and were more pathogenic in mice than the corresponding viruses with truncated (232 amino acids) PA-X proteins [30].

Since IAV NS1 and PA-X proteins are virulence factors that modulate host innate immune responses, the interplay between both viral proteins is important for viral replication and pathogenicity. We have shown that regulation of innate immune responses by IAV NS1 and PA-X proteins determines virus fitness and pathogenesis in vitro and in vivo, respectively [12,35,36,37]. In addition, we have shown a functional co-evolution of NS1 and PA-X proteins in pH1N1 [36]. Here, we evaluated the fitness and pathogenicity of recombinant IAV encoding PA or NS1 genes from old (1996–1997) or recent (since 2016) H5N1 strains. Consistent with our previous findings [36,37], our studies suggest the importance of a co-evolution in the regulation of host gene expression by H5N1 NS1 and PA-X proteins, with effects on viral fitness and pathogenesis.

## 2. Materials and Methods

### 2.1. Cell Lines

Madin-Darby canine kidney (MDCK, ATCC CCL-34), human lung epithelial carcinoma A549 (ATCC CCL-185), human embryonic kidney (HEK293T, ATCC CRL-11268), and chicken embryo fibroblast DF-1 (ATCC CRL-12203) cells were grown in Dulbecco’s modified Eagle’s medium (DMEM; Mediatech, Inc, Manassas, VA, USA) containing 10% fetal bovine serum (FBS) and 2 mM L-glutamine at 37 °C in a 5% CO_2_ incubator.

### 2.2. Analysis of NS1 and PA-X Sequences

Human and avian H5N1 PA-X and NS1 sequences available at the Influenza Research Database (https://www.fludb.org/ (accessed on 23 June 2020)) were downloaded, introducing in the search the time periods shown in Table 1, Table 2, Table 3 and Table 4, and either human or avian origin. The sequences were aligned using the “analyze sequence variation (SNP)” function available in this webpage.

### 2.3. Plasmids

The NS1 and PA-X genes fused to an N-terminal HA epitope tag were cloned into the pCAGGS HA-NH_2_ plasmid [38,39] using SphI and NheI restriction sites. The amino acid sequences were as follows: for PA-X_OR_, A/Hong Kong/483/1997, H5N1 strain, Pubmed protein ID: AAF74340.1; for PA-X_CIR_, A/mallard/Italy/3401/2005, H5N1 strain, Pubmed protein ID: AGJ72962.1; for NS1_OR,_ A/Hong Kong/156/97, H5N1 strain, Pubmed protein ID: O56264.1; and for NS1_CIR,_ A/duck/Bangladesh/27820/2015, H5N1 strain, Pubmed protein ID: AQY17641.1.

To obtain the pDZ-NS_CIR_ and pDZ-NS_OR_ rescue plasmids, the coding regions of NS1_OR_ and NS1_CIR_ were cloned into a pDZ plasmid carrying non-overlapping NS1 and NEP genes (pDZ-NSs) using the porcine teschovirus 2A autoproteolytic cleavage site, as previously described [40]. Rescue plasmids encoding the PA_OR_ and PA_CIR_ proteins were obtained by cloning PA_OR_ and PA_CIR_ viral segments into the pHW2000 plasmid using BsmBI restriction sites.

### 2.4. Effect of NS1 and PA-X Proteins on Gene Expression

HEK293T or DF-1 cells (96-well plate format, 5 × 10^4^ cells/well, triplicates) were transiently co-transfected, with 200 ng/well of pCAGGS plasmids encoding either NS1 and/or PA-X OR or CIR proteins fused to an N-terminal HA tag, or transfected with empty plasmid as internal control, together with 20 ng/well of a pCAGGS plasmid encoding Renilla luciferase (Rluc), using Lipofectamine 3000 (Thermo Fisher Scientific, Waltham, MA, USA). At 24 h post-transfection (h p.t), cells were lysed using passive lysis buffer (Promega, Madison, WI, USA), and Rluc activity was measured using a Biolux *renilla* luciferase reagent (New England BioLabs, Ipswich, MA, USA) and a Lumicount luminometer (Apliskan, Thermo Scientific, Waltham, MA, USA). The means and standard deviations were calculated and a two-tailed Student *t* test was used for statistical analysis using GraphPad Prism software (v8).

### 2.5. Effect of NS1 and PA-X Proteins on IFN Induction after SeV Infection

To evaluate the effect of H5N1 IAV NS1 and PA-X proteins on induction of IFN responses, HEK293T cells (96-well plate format, 5 × 10^4^ cells/well, triplicates) were transiently co-transfected with 100 ng/well of pCAGGS plasmids encoding OR or CIR HA-tagged NS1 and/or PA-X proteins, or empty plasmid as control, together with 50 ng/well of a plasmid expressing firefly luciferase (Fluc) under the control of the IFN-sensitive response element (ISRE) promoter (pISRE-Fluc) [41], together with 20 ng/well of a pCAGGS plasmid encoding Rluc, using calcium phosphate (Agilent Technologies, Santa Clara, CA, USA). At 24 h p.t, cells were washed with PBS and infected with a multiplicity of infection (MOI) of 3 with the Sendai virus (SeV), strain Cantell, as previously described [41]. At 24 h post-infection (h p.i), cells were lysed using passive lysis buffer (Promega). Luciferase expression in the cell lysates was determined using a dual-Glo luciferase kit (Promega) according to the manufacturer’s instructions. Measurements were recorded with a Lumicount luminometer (Apliskan, Thermo Scientific), and the mean values and standard deviations were calculated. Statistical analysis was performed using a two-tailed Student *t* test and GraphPad Prism software (v8).

### 2.6. Analysis of NS1 and PA-X Expression by Western Blot

For Western blotting, transfected cells were lysed in buffer containing 100 mM Tris-HCl (pH 6.8), 4% SDS, 20% glycerol, 0.2% bromophenol blue, and 20% β-mercaptoethanol and boiled for 5 min. Then, proteins in cell lysates were separated by SDS-PAGE and transferred onto nitrocellulose membranes. Subsequently, membranes were blocked for 1 h in PBS containing 5% dried skim milk and 0.1% Tween-20 and incubated with an anti-HA polyclonal antibody (Sigma, Stockholm, Sweden) or an anti-actin monoclonal antibody (Sigma) at 4 °C overnight. Horseradish peroxidase (HRP) secondary antibodies (Merck life science, Darmstadt, Germany) specific for either mouse or rabbit immunoglobulins (Ig) were used to detect bound primary antibodies. Proteins in the membranes were detected with a SuperSignal West Femto maximum-sensitivity chemiluminescent substrate kit (Thermo Scientific) following the manufacturer’s instructions.

### 2.7. Generation of Recombinant Viruses

Recombinant A/Puerto Rico/8/1934 (PR8) H1N1 viruses were generated as previously described [40,42,43,44]. Briefly, cocultures (1:1) of HEK293T and MDCK cells (6-well plate format, 1 × 10^6^ cells/well, triplicates) were transiently co-transfected in suspension using polyethileneimine (PEI; Polysciences, Warrington, PA, USA) with 1 μg of each of the six ambisense pHW2000 PR8 WT plasmids (pHW-PB2, -PB1, -HA, -NP, -NA, and -M), pDZ-NSs plasmids encoding NS1_OR_ or NS1_CIR_, and the pHW2000 plasmids encoding PA_OR_ or PA_CIR_ [36]. At 12 h p.t, the medium was replaced with DMEM containing 0.3% bovine serum albumin (BSA), and 0.5 μg/mL of tosylsulfonyl phenylalanyl chloromethyl ketone (TPCK)-treated trypsin (Sigma). At 96 h p.t, tissue culture supernatants were collected and used to infect fresh monolayers of MDCK cells. At 3 days post-infection (dpi), recombinant viruses were plaque purified and virus stocks were prepared in confluent monolayers of MDCK cells. Viral stocks were titrated by immunofocus assay (focus-forming units [FFU]/mL) on MDCK cells [45]. MDCK cells (96-well plate format, 5 × 10^4^ cells/well, triplicates) were infected with 10-fold serial dilutions of the tissue culture supernatants. After 8 h of viral infection at 37 °C, cells were fixed and permeabilized with PBS containing 4% formaldehyde and 0.1% Triton X-100 for 20 min at room temperature. Then, cells were incubated in a blocking solution (PBS with 2.5% BSA) for 1 h at room temperature and subsequently incubated with the anti-NP monoclonal antibody (MAb) HB-65 (ATCC H16-L10-4R5) for 2 h at 37 °C and washed with PBS. Cells were then incubated at 37 °C for 1 h with a fluorescein isothiocyanate (FITC)-conjugated rabbit anti-mouse IgG secondary antibody (Life Technologies; Carlsbad, CA, USA). Viral titers (FFU/mL) were quantified by counting all the cells that were positive for NP expression in the triplicate wells, and the means and standard deviations were calculated as previously described [45]. Experiments involving infectious viruses were performed at the biosafety level 3 (BSL3) and animal (ABSL3) facilities in the Animal Health Research Center (INIA-CISA), Madrid (Spain).

### 2.8. Virus Growth Kinetics

Confluent monolayers of canine MDCK, human A549 or avian DF-1 cells (4 × 10^5^ cells/well, 12-well plates, triplicates) were infected (MOI of 0.001 for MDCK cells; MOI 0.01 for A549 and DF-1 cells), for 1 h at room temperature, and incubated in DMEM media supplemented with 0.3% BSA and 1 (MDCK) or 0.5 (A549 and DF-1) μg/mL TPCK-treated trypsin, at 37 °C. Tissue culture supernatants collected at 24, 48, and 72 h p.i were titrated on MDCK cells (96-well plates, 5 × 10^4^ cells/well, triplicates) by immunofluorescence (FFU/mL), as described above. The means and standard deviations were calculated, and statistically significant differences were determined using a two-tailed Student *t* test and GraphPad Prism software (v8).

### 2.9. Pathogenesis of Recombinant Viruses in Mice

Seven-to-nine-week-old wild-type (WT) A129 mice were bred in the animal care facility of the Department of Animal reproduction at INIA and housed under pathogen-free conditions at the biosafety level 3 (BSL3) animal facilities in the Animal Health Research Center (INIA-CISA), Madrid (Spain). Animal experimental protocols were approved by the Ethical Review Committee at the INIA-CISA and Comunidad de Madrid (Permit number: PROEX 116/19), in strict accordance with EU guidelines 2010/63/UE about the protection of animals used for experimentation and the Spanish Animal Welfare Act 32/2007. For viral inoculations, mice were first anesthetized intraperitoneally (i.p.) with 100 mg/kg of body weight of ketamine (La Casa del Campo, Cádiz, Spain) and 5/mg/kg of xylacin (La Casa del Campo) and then infected intranasally (i.n.) with 30 μL of the indicated recombinant viruses. Mice (*N* = 5/group) were examined each day for weight loss, clinical symptoms (such as malaise, respiratory distress, and lack of movement), and mortality. Percent body weight loss was determined relative to the starting weight. Mice losing more than 25% of their initial body weight were considered to have reached the experimental endpoint and were humanely euthanized. In another set of experiments, mice (*N* = 4/group) were infected as described above and, at 2 and 4 dpi, animals were euthanized and lungs were extracted. The right and left lobules were used for analyzing viral titers or for extracting the RNA, respectively. For viral titers, the right lobules were transferred to tubes containing 1 mL of DMEM and one 5 mm stainless steel bead (Qiagen, Hilden, Germany) and homogenized using the TissueLyser II (Qiagen) at 25 Hz for 5 min, followed by centrifugation at 5000× *g* for 10 min at 4 °C. Viral titers were determined by immunofluorescence assay (FFU/mL) as outlined above.

The left lobules were collected and submerged into RNAlater (Thermo Fisher Scientific), incubated overnight at 4 °C and then stored at −80 °C until processing. For RNA extraction, tissue samples were transferred to tubes containing one 5 mm stainless steel bead (Qiagen) and 1 mL of RLT buffer (Qiagen) with β-mercaptoethanol. The samples were homogenized using the TissueLyser II (Qiagen) at 25 Hz for 5 min, followed by centrifugation at 5000× *g* for 10 min at 4 °C. Then, total RNAs were purified using an RNeasy mini kit (Qiagen) following the manufacturer’s instructions. Reverse transcriptase (RT) reactions were conducted with a high-capacity cDNA transcription kit (Applied Biosystems, Waltham, MA, USA) at 37 °C for 2 h. Quantitative PCRs (qPCRs) were performed using TaqMan gene expression assays (Applied Biosystems) specific for the mRNA of chemokine (C-C) motif ligand 2 (CCL2, Mm00441242_m1), TNF (Mm00443258_m1), and IFN-induced protein with tetratricopeptide repeats 2 (IFIT2, Mm00492606_m1). The 2^−ΔΔCT^ method was used for quantification, and values were presented as fold induction [46]. For virus titers and qPCR results, one-way analysis of variance (ANOVA) was performed using GraphPad Prism software (v8).

## 3. Results

### 3.1. Identification and Selection of Amino Acid Changes in H5N1 IAV NS1 and PA-X Proteins

**Figure 1:**
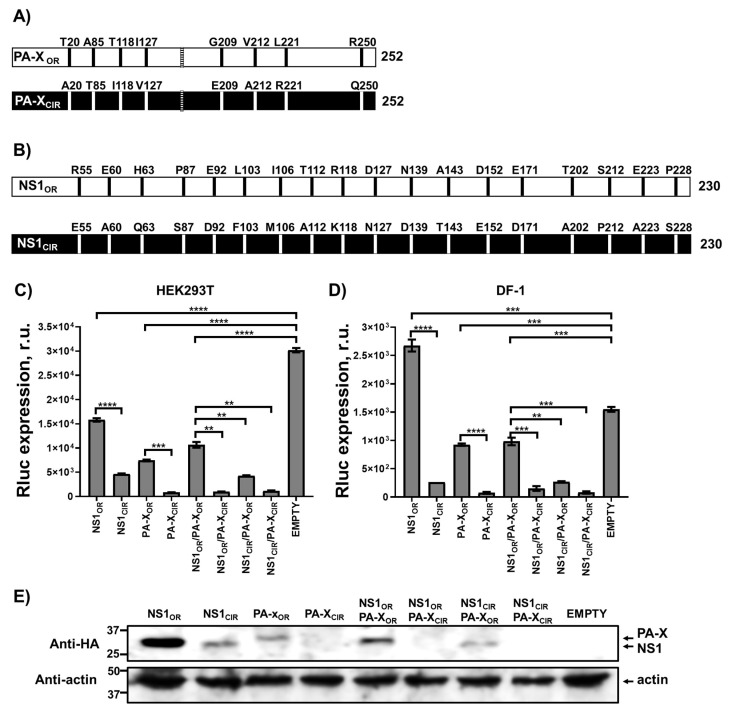
Effect of H5N1 NS1 and PA-X amino acid changes on host gene expression. (**A**,**B**) Schematic representation of PA-X (**A**) and NS1 (**B**) amino acid residues most frequently present in H5N1 IAV circulating in avian and humans during 1996–1997 (OR, original), and from viruses circulating since 2016 (CIR, circulating). The PA-X +1 frameshift motif (UCC UUU CGU C) is indicated with a striped bar (**A**). (**C**–**E**) Human HEK293T (**C**,**E**) and avian DF-1 (**D**) cells were transiently co-transfected with a pCAGGS empty plasmid, or with pCAGGS plasmids expressing the indicated HA epitope-tagged NS1 and PA-X viral proteins, together with a Rluc expressing pCAGGS plasmid. NS1_CIR_ protein encodes the amino acid changes R55E, E60A, H63Q, P87S, E92D, L103F, I106M, T112A, R118K, D127N, N139D, A143T, D152E, E171D, T202A, S212P, E223A, and P228S; whereas the PA-X_CIR_ protein encodes the amino acid changes T20A, A85T, T118I, I127V, G209E, V212A, L221R, and R250Q. At 24 h p.t, Rluc expression (**C** and **D**) was quantified using a luminometer. Error bars represent the standard deviations from triplicates. *, *p* < 0.05; **, *p* < 0.005; ***, *p* < 0.0005; ****, *p* < 0.0001 (NS1_OR_ versus NS1_CIR_, PA-X_OR_ versus PA-X_CIR_, NS1_OR_/PA-X_OR_ versus NS1_OR_/PA-X_CIR_, NS1_OR_/PA-X_OR_ versus and NS1_CIR_/PA-X_OR_ and NS1_OR_/PA-X_OR_ versus NS1_CIR_/PA-X_CIR_) using a Student’s *t*-test. R.u., relative units. NS1, PA-X, and actin protein expression levels in HEK293T transfected cells were analyzed by Western blot using cells extracts and antibodies specific for the HA tag, to detect NS1 and PA-X proteins (bottom and top bands in the upper blot, respectively); and actin (bottom blot) (**E**). Molecular mass markers (in kDa) are indicated on the left. The experiments were repeated three times, with similar results.

We previously showed that the pH1N1 IAV that emerged in humans in 2009, has evolved to incorporate amino acid changes in the NS1 and PA-X proteins leading to increased and decreased shutoff activity, respectively [36,42]. To analyze whether the NS1 and PA-X proteins of other IAV subtypes also evolve over time, the sequences of NS1 and PA-X proteins of H5N1 IAV circulating in avian and human hosts during 1996–1997 (named as OR, from original), when the first avian and human cases were reported, were compared to the NS1 and PA-X proteins of H5N1 IAV circulating since 2016 (named as CIR, from circulating). No changes were observed in the PA frameshift sequence between OR and CIR H5N1 strains. However, eight amino acid changes (T20A, A85T, T118I, I127V, G209E, V212A, L221R, and R250Q) were observed in the most frequent sequences of PA-X (Figure 1A), whereas 18 amino acid changes (R55E, E60A, H63Q, P87S, E92D, L103F, I106M, T112A, R118K, D127N, N139D, A143T, D152E, E171D, T202A, S212P, E223A, and P228S) were identified in the most frequent sequences of NS1 (Figure 1B). In addition, a 5 amino acid deletion, between amino acids 80 and 84, leading to a 225 amino acid long NS1 protein, was observed in most of the viruses isolated from 2001 to 2015. However, since, in this work, we studied the consensus sequence from NS1 proteins from H5N1 viruses isolated in 1996–1997 and since 2016, both proteins used in this study were 230 amino acid in length (Figure 1B).

We analyzed the prevalence of these mutations over time, showing the percentage of sequences encoding the different amino acids at each particular position in H5N1 PA-X (Table 1 and Table 2) and NS1 (Table 3 and Table 4) proteins. For PA-X, amino acid changes T20A, A85T, T118I, I127V, G209E, V212A, and R250Q, were selected rapidly in avian viruses (Table 1), since more than 90% of the sequences isolated during 1998–2000 contain the amino acids encoded by the viruses preferentially circulating nowadays. However, although the amino acid changes T20A and G209E were selected quickly in avian viruses, a mixture of isolates containing T and A at position 20, and G and E residues at position 209 was present in the period of 2016–2020 (Table 1). The amino acid change L221R was selected later, with most of the H5N1 IAV isolates from 2001 to 2005 encoding the residue circulating nowadays (Table 1). Notably, H5N1 IAV containing the residues most common in avian isolates were also the ones most frequently infecting humans (Table 2), as could be expected taking into account that most of the infections in humans occur in subjects in close contact with infected avian species [47]. In H5N1 IAV isolated from humans, PA-X residues T20, A85, T118, G209, V212, L221, and Q250 were present in viruses infecting humans during 1996–1997. The residues I and V at position 127 were present at 42 and 58% of the H5N1 viruses infecting humans during 1996–1997. However, in recent years, the most frequent residues were T85, I118, V127, E209, A212, R221, and Q250, and a mixture of A and T at amino acid 20 (Table 2). These data suggest that most of the changes in the PA-X protein of H5N1 viruses are likely beneficial as they became fixed at the global level. However, whereas the amino acid changes G209E, V212A, L221R, and R250Q, encode silent mutations to the PA protein, the amino acid changes T20A, A85T, T118I, and I127V also affect the PA sequence, making difficult a priori to discern whether these changes were selected because they are beneficial for the PA-X and/or for the PA protein.

For the NS1 protein, most of the amino acid changes were selected quickly in avian H5N1 viruses; however, interestingly, there was a high variability in the amino acids identified in these H5N1 IAV during the time frames analyzed (Table 3). There are some amino acid positions (e.g., 112, 127, 171, and 212) in which different residues were selected over time (Table 3). Furthermore, we noted that there was an amino acid change at residues 92 and 139 (E92D and N139D, respectively) in H5N1 viruses isolated from 1998 to 2015 in avian hosts. However, since 2016, an important variability was observed in these residues (Table 3). Sequences obtained from H5N1 viruses isolated in humans showed less variability (Table 4). Nevertheless, we observed that different residues were selected over time at positions 112, 127, 143, and 212 (Table 4). This analysis suggests a high complexity in the evolution of these virulence factors, which is most clear in H5N1 viruses isolated from avian species, the natural host. Importantly, since avian hosts include multiple species of wild birds, or poultry, the observed variability could be linked to differences between hosts and/or their innate immune systems. For that reason, evaluating the functional consequences of the observed changes is highly important.

**Table 1:** Amino acid changes in PA-X proteins from H5N1 IAV isolated in avian.

Position	Amino Acid	1996–1997	1998–2000	2001–2005	2006–2010	2011–2015	2016–2020
20	T	40.7	0	0.93	1.59	70.77	31.86
	A	59.3	100	98.75	97.97	29.09	68.13
	Other	0	0	0.31	0.42	0.13	0
85	A	40.74	0	0.61	0.62	1.85	1.44
	T	59.3	100	99.23	99.26	98.14	98.56
	Other	0	0	0.15	0.1	0	0
118	T	42.86	0	0.77	0	0	23.7
	I	57.14	100	99.22	99.68	93.84	70.19
	Other	0	0	0	0.31	6.15	6.73
127	I	25	0	0.46	0.42	0.26	0
	V	75	100	99.38	99.37	99.73	98.56
	Other	0	0	0.62	0.21	0	1.44
209	G	46.42	0	0.92	0.42	1.04	24.88
	E	53.57	100	98.92	99.48	98.29	75.11
	Other	0	0	0.15	0.1	0.66	0
212	V	39.28	0	1.4	2.6	1.44	0
	A	57.15	92	96.9	97.29	98.16	99.04
	Other	3.57	8	1.7	0.1	0.39	0.95
221	L	50	68	1.69	0.21	0.26	0.95
	R	50	32	97.69	99.58	99.6	98.08
	Other	0	0	0.62	0.21	0.13	0.95
250	R	46.43	4	6.33	0.52	1.7	1.91
	Q	46.43	96	92.73	98.84	97.37	95.21
	Other	7.14	0	0.93	0.63	0.92	2.87

Frequencies of identified mutations in H5N1 IAV PA-X proteins isolated from avian at the indicated time periods. Publicly available sequences in the Influenza Research Database (https://www.fludb.org/brc/home.spg?decorator=influenza (accessed on 23 June 2020)) were downloaded and the frequencies represented according to the year of isolation. The original amino acid present in 1996–1997 H5N1 IAV isolates is depicted first, whereas the amino acid most frequently found nowadays is depicted second. The numbers of sequences available at the Influenza Research Database are 27, 25, 644, 939, 739, and 204 sequences, for periods 1996–1997, 1998–2000, 2001–2005, 2006–-2010, 2011–2015, and 2016–2020, respectively.

**Table 2:** Amino acid changes in PA-X proteins from H5N1 IAV isolated in humans.

Position	Amino Acid	1996–1997	1998–2000	2001–2005	2006–2010	2011–2015	2016–2020
20	T	100	ND	0	1.35	50	ND
	A	0	ND	100	98.65	50	ND
85	A	100	ND	0	2.7	0	ND
	T	0	ND	98.71	97.3	100	ND
	Other	0	ND	1.28	0	0	ND
118	T	100	ND	0	0	0	ND
	I	0	ND	100	100	100	ND
127	I	42.1	ND	0	0	0	ND
	V	57.89	ND	100	100	100	ND
	Other	0	ND	0	1.34	0	ND
209	G	100	ND	1.26	2	0	ND
	E	0	ND	98.73	98	100	ND
212	V	100	ND	1.26	0.67	0	ND
	A	0	ND	98.73	99.32	100	ND
221	L	100	ND	0	0	0	ND
	R	0	ND	98.73	100	100	ND
	Other	0	ND	1.26	0	0	ND
250	R	0	ND	1.26	0	3.57	ND
	Q	100	ND	98.73	100	96.43	ND

Frequencies of identified mutations in H5N1 IAV PA-X proteins isolated from humans at the indicated time periods. Publicly available sequences in the Influenza Research Database (https://www.fludb.org/brc/home.spg?decorator=influenza (accessed on 23 June 2020)) were downloaded and the frequencies represented according to the year of isolation. The original amino acid present in 1996–1997 H5N1 IAV isolates is depicted first, whereas the amino acid most frequently found nowadays is depicted second. The numbers of sequences available at the Influenza Research Database are 19, 0, 77, 148, 28, and 0 sequences for periods 1996–1997, 1998–2000, 2001–2005, 2006–2010, 2011–2015, and 2016–2020, respectively.

**Table 3:** Amino acid changes in NS1 proteins from H5N1 IAV isolated in avian.

Position	Amino Acid	1996–1997	1998–2000	2001–2005	2006–2010	2011–2015	2016–2020
55	R	75	65.38	1.5	0.84	0.32	3.04
	E	25	34.62	96.98	94.1	87.93	96.34
	Other	0	0	1.5	5.67	11.74	1.83
60	E	70	65.38	1.5	2.22	0.54	3
	A	30	34.62	9.39	97.21	80.54	87.95
	Other	0	0	0.1	0.55	18.91	9
63	H	60	3.84	1.07	0.18	0	2.41
	Q	25	30.77	98.06	99.81	98.91	94.57
	Other	15	65.38	0.86	0	1.08	3.01
87	P	75	65.38	3.11	0.83	2.17	4.89
	S	25	34.61	93.23	98.98	97.5	96.34
	Other	0	0	3.65	0.18	0.32	0
92	E	55	0	0.43	0.092	44.45	37.35
	D	45	100	99.46	99.91	55.21	62.65
	Other	0	0	0.1	0	0.32	0
103	L	60	3.8	1.18	0.092	0	2.4
	F	25	26.92	97.42	97.4	99.78	94.57
	Other	15	69.23	1.39	2.5	0.21	3
106	I	60	11.53	1.29	0.28	0	0
	M	40	84.61	98.71	99.72	100	100
	Other	0	3.8	0	0	0	0
112	T	70	65.38	7.95	48.84	6.1	5.42
	A	30	34.61	91.73	44	13.49	54.82
	Other	0	0	0.32	7.24	80.41	39.76
118	R	85	15.38	6.87	1.95	4.13	40.96
	K	15	84.61	93.12	98.05	95.86	59.03
127	D	55	0	0.43	0	0	0
	N	30	38.46	18.8	2.78	3.99	53
	Other	15	61.54	80.77	97.21	95.32	46.99
139	N	65	7.69	1.93	1.2	2.61	69.88
	D	35	92.3	96.88	96.75	90.53	29.51
	Other	0	0	1.18	2	6.85	0.6
143	A	65	3.84	0.86	0.37	0.11	0
	T	35	96.15	96.45	91.37	92.93	99.4
	Other	0	0	2.68	8.25	7.07	0.6
152	D	55	7.69	1.07	0.092	0.32	1.2
	E	45	92.31	98.17	98.98	99.02	98.8
	Other	0	0	0.75	0.93	0.64	0
171	E	55	0	0.75	0.27	0	0
	D	20	38.46	28.76	83.22	8.27	62.65
	Other	25	61.54	70.49	93.21	91.73	37.35
202	T	55	0	0.86	1.76	1.63	0
	A	45	100	97.85	97.86	98.14	100
	Other	0	0	0.96	0.37	0.22	0
212	S	55	0	1.61	0.092	0	0.6
	P	45	100	22.31	5	12.33	43.97
	Other	0	0	76.07	94.9	87.66	55.42
223	E	61.1	0	0.54	0.75	0.11	0
	A	38.89	100	99.46	99.25	99.89	100
228	P	61.1	0	2	3.11	1.21	6
	S	38.89	100	97.73	93.69	98.79	94
	Other	0	0	0.21	0.092	0	0

Frequencies of identified mutations in H5N1 IAV NS1 proteins isolated from avian at the indicated time periods. Publicly available sequences in the Influenza Research Database (https://www.fludb.org/brc/home.spg?decorator=influenza (accessed on 23 June 2020)) were downloaded and the frequencies represented according to the year of isolation. The original amino acid present in 1996–1997 H5N1 IAV isolates is depicted first, whereas the amino acid most frequently found nowadays is depicted second. The numbers of sequences available at the Influenza Research Database are 20, 36, 930, 1074, 920, and 164, for periods 1996–1997, 1998–2000, 2001–2005, 2006–2010, 2011–2015, and 2016–2020, respectively.

**Table 4:** Amino acid changes in NS1 proteins from H5N1 IAV isolated in humans.

Position	Amino Acid	1996–1997	1998–2000	2001–2005	2006–2010	2011–2015	2016–2020
55	R	100	ND	0	0	0	ND
	E	0	ND	98.68	97.33	87.09	ND
	Other	0	ND	1.32	2.66	12.9	ND
60	E	100	ND	0	0	0	ND
	A	0	ND	100	97.33	100	ND
	Other	0	ND	0	2.66	0	ND
63	H	100	ND	0	0	0	ND
	Q	0	ND	100	100	100	ND
87	P	91.3	ND	1.31	0	3.22	ND
	S	8.7	ND	98.68	100	96.77	ND
92	E	100	ND	0	0	3.22	ND
	D	0	ND	100	100	96.77	ND
103	L	100	ND	0	0	0	ND
	F	0	ND	100	100	100	ND
106	I	100	ND	0	0	0	ND
	M	0	ND	100	100	100	ND
112	T	100	ND	1.33	11.33	9.68	ND
	A	0	ND	98.68	86	35.48	ND
	Other	0	ND	0	2.67	54.84	ND
118	R	100	ND	0	0	0	ND
	K	0	ND	100	100	100	ND
127	D	89.47	ND	0	0	0	ND
	N	11.76	ND	1.31	0.66	0	ND
	Other	0	ND	98.68	99.33	100	ND
139	N	100	ND	0	0	0	ND
	D	0	ND	100	96.66	70.96	ND
	Other	0	ND	0	3.33	29.03	ND
143	A	100	ND	0	0	0	ND
	T	0	ND	97.37	98	64.51	ND
	Other	0	ND	2.63	2	35.49	ND
152	D	100	ND	0	0	0	ND
	E	0	ND	100	99.33	100	ND
	Other	0	ND	0	0.66	0	ND
171	E	94.73	ND	0	0	0	ND
	D	5.26	ND	3.94	0	0	ND
	Other	0	ND	96.05	100	100	ND
202	T	100	ND	0	0	0	ND
	A	0	ND	100	95.33	100	ND
	Other	0	ND	0	4.67	0	ND
212	S	89.47	ND	0	0	0	ND
	P	10.52	ND	3.94	1.33	29	ND
	Other	0	ND	96.05	98.67	71	ND
223	E	100	ND	0	0.68	0	ND
	A	0	ND	100	99.32	100	ND
228	P	100	ND	0	1.36	3.22	ND
	S	0	ND	100	98.64	96.77	ND

Frequencies of identified mutations in H5N1 IAV NS1 proteins isolated from hum at the indicated time periods. Publicly available sequences in the Influenza Research Database (https://www.fludb.org/brc/home.spg?decorator=influenza (accessed on 23 June 2020)) were downloaded and the frequencies represented according to the year of isolation. The original amino acid present in 1996–1997 H5N1 IAV isolates is depicted first, whereas the amino acid most frequently found nowadays is depicted second. The numbers of sequences available at the Influenza Research Database are 23, 0, 76, 150, 31, and 0 sequences for periods 1996–1997, 1998–2000, 2001–2005, 2006–2010, 2011–2015, and 2016–2020, respectively. ND: 0 sequences available in the Influenza Research Database.

### 3.2. Increased NS1 and PA-X Shutoff Activity in Currently Circulating H5N1 IAV

The NS1 proteins of some influenza strains induce cellular shutoff by blocking the maturation of RNA polymerase II transcripts and the nuclear export of host mRNAs, a mechanism mediated by its ability to bind to the cleavage and polyadenylation specific factor 30 (CPSF30) host protein [19,41,48,49], and to the poly(A)-binding protein II (PABII) [18]. Additionally, influenza PA-X selectively degrades mRNAs transcribed by host RNA polymerase II [27,31,36,50,51,52]. In addition, we and others have been able to demonstrate in previous studies how the inhibition of reporter gene expression (e.g., Luciferase) in cells expressing influenza NS1 and PA-X variants correlate with the levels of inhibition of real host genes including IFN and IFN-regulated genes [36,37,43,44,50,53,54,55]. Therefore, to analyze whether the amino acid changes in PA-X and/or NS1 affect the ability of the H5N1 viral proteins to mediate cellular shutoff, human HEK293T (Figure 1C) and avian DF-1 (Figure 1D) cells were transiently co-transfected with a pCAGGS expression plasmid encoding Renilla luciferase (Rluc), together with pCAGGS plasmids encoding HA epitope-tagged NS1_OR_ and PA-X_OR_ genes_,_ referring to the proteins from H5N1 viruses preferentially circulating during 1996–1997; or NS1_CIR_ and PA-X_CIR_ genes, referring to proteins from H5N1 viruses preferentially circulating nowadays; alone or in combinations. All these genes were transcribed by the host RNA polymerase II using this experimental approach. At 24 h p.t, levels of Rluc expression were determined (Figure 1C,D). H5N1 NS1_OR_ did not efficiently inhibit host gene expression, as determined by levels of Rluc expression in both HEK293T or avian DF-1 cells (Figure 1C,D, respectively), whereas H5N1 NS1_CIR_ efficiently blocked host gene expression in both cell lines (Figure 1C,D). These results were not surprising, as it was previously shown that the NS1 protein of H5N1 IAV circulating during 1996–1997 contain amino acids L103 and I106, leading to inefficient host gene inhibition; whereas amino acid substitutions F103 and M106, present in the NS1 from H5N1 IAV circulating since 1998, and therefore present in the NS1_CIR_, result in proteins with increased ability to block host gene expression [24,25].

Similarly, H5N1 PA-X_CIR_ inhibited host gene expression around 10-fold more efficiently than PA-X_OR_, as determined by Rluc expression in HEK293T (Figure 1C) and DF-1 (Figure 1D) cells. When expressed together, NS1_CIR_ and PA-X_CIR_ were the most efficient at inhibiting host gene expression, whereas NS1_OR_ and PA-X_OR_ had the least effect on inhibition of reporter gene expression in either human or avian cells (Figure 1C,D, respectively). To further validate these findings, and, since NS1 and PA-X inhibit their own expression when expressed from plasmids under the control of a polymerase II promoter [37,41,56], we next evaluated H5N1 NS1 and PA-X protein expression levels in HEK293T cells by Western blot (Figure 1E). Notably, when expressed alone, NS1_OR_ and PA-X_OR_ were expressed to higher levels than NS1_CIR_ and PA-X_CIR_, respectively (Figure 1E). When simultaneously expressed, only H5N1 NS1 proteins were detected by Western blot. This is likely due to the lower levels of PA-X expression detected by Western blot when expressed alone (Figure 1E). Unfortunately, we did not detect H5N1 PA-X or NS1 protein expression in DF-1 cells, most likely because of the lower transfection efficiency compared to HEK293T cells, as shown by the lower levels of Rluc expression in DF-1 cells compared to HEK293T cells (Figure 1C,D). These data indicate that the NS1 and PA-X proteins from currently circulating H5N1 IAV possess increased abilities to inhibit host gene expression compared with H5N1 viruses circulating during 1996–1997.

### 3.3. Effect of H5N1 IAV NS1 and PA-X Amino Acid Changes on IFN Responses Induced by SeV Infection

**Figure 2:**
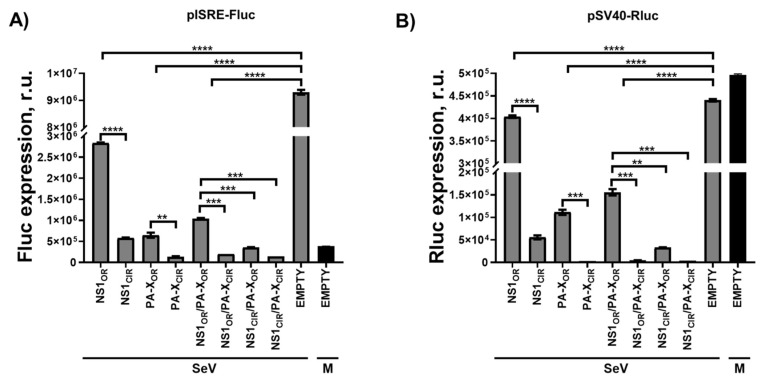
Effect of H5N1 NS1 and PA-X amino acid changes on IFN responses: Human HEK293T cells were transiently co-transfected with a pCAGGS empty plasmid control, or with pCAGGS plasmids expressing the indicated HA epitope-tagged NS1 and PA-X viral proteins, together with a plasmid expressing Fluc under an ISRE dependent promoter, and a plasmid constitutively expressing Rluc. At 24 h p.t, cells were infected with SeV (Cantell strain, MOI 3) or left mock-infected (M), and 20 h later expression of Fluc (**A**) and Rluc (**B**) was analyzed by luminescence. Data show the mean and standard deviations from cells in triplicate. Experiments were repeated 3 times in triplicate, with similar results. *, *p* < 0.05; **, *p* < 0.005; ***, *p* < 0.0005; ****, *p* < 0.0001 (NS1_OR_ versus NS1_CIR_, PA-X_OR_ versus PA-X_CIR_, NS1_OR_/PA-X_OR_ versus NS1_OR_/PA-X_CIR_, NS1_OR_/PA-X_OR_ versus NS1_CIR_/PA-X_OR_, and NS1_OR_/PA-X_OR_ versus NS1_CIR_/PA-X_CIR_) using Student’s *t* test (*n* = 3 per time point). R.u., relative units.

H5N1 NS1 and PA-X proteins are virulence factors that have been shown to modulate innate immune responses [12,29]. To investigate whether the H5N1 NS1 and PA-X protein variants differentially modulate innate immune responses in the context of viral infection, human HEK293T cells were co-transfected with pCAGGS plasmids expressing the H5N1 NS1 and PA-X variants, alone or in combinations, together with reporter plasmids expressing Firefly luciferase (Fluc) under the control of an IFN-stimulated response element (ISRE) promoter (Figure 2A), and a pCAGGS plasmid expressing Rluc (Figure 2B). At 24 h p.t, cells were mock-infected or infected with SeV, Cantell strain, at a multiplicity of infection (MOI) of 3. ISRE promoter activation was evaluated 24 h p.i by assessing Fluc expression levels (Figure 2A). Furthermore, constitutive levels of Rluc (Figure 2B) were determined. Reflecting IFN induction, high levels of Fluc expression driven by the ISRE promoter were observed after SeV infection in cells transfected with the pCAGGS empty plasmid (Figure 2A). As expected, activation of ISRE promoter was significantly reduced in cells transfected with H5N1 PA-X and/or NS1 proteins (Figure 2A). However, differences in the activation of ISRE promoter were detected for the different H5N1 NS1 and PA-X variants. Activation of the ISRE promoter was less efficient in cells transfected with the plasmids expressing H5N1 NS1_CIR_ and PA-X_CIR_ variants than in cells transfected with the plasmids expressing H5N1 NS1_OR_ and PA-X_OR_, respectively (Figure 2A). Notably, shutoff activity for H5N1 NS1 and PA-X variants, as measured by Rluc levels, showed higher Rluc expression inhibition for H5N1 NS1_CIR_ than for NS1_OR_, and for H5N1 PA-X_CIR_ than for PA-X_OR_ (Figure 2B). These results were similar to those observed for Rluc expression in the absence of viral infection (Figure 1C). These data indicate that the NS1 and PA-X proteins from H5N1 IAV circulating nowadays have evolved to increase their ability to inhibit gene expression and that this shutoff activity likely affects IFN-related transcripts.

### 3.4. Identification of H5N1 IAV PA-X Amino Acid Residues Involved in the Ability to Inhibit Host Gene Expression and IFN Response

**Figure 3:**
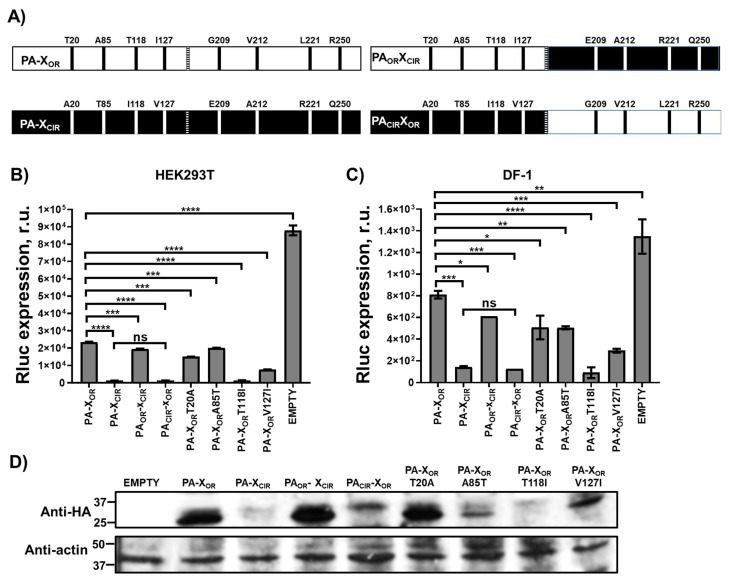
Effect of H5N1 IAV PA-X amino acid changes on host gene expression. (**A**) Schematic representation of H5N1 IAV PA-X WT and chimeric constructs. (**B**,**C**) Human HEK293T (**B**) and avian DF-1 (**C**) cells were transiently co-transfected with an empty pCAGGS plasmid control, or with pCAGGS plasmids expressing the indicated HA epitope-tagged H5N1 PA-X constructs, together with Rluc-expressing plasmid. PA-X_CIR_ protein encodes the amino acid changes T20A, A85T, T118I, I127V, G209E, V212A, L221R, and R250Q. PA_OR_-X_CIR_ encodes the amino acid changes G209E, V212A, L221R, and R250Q. PA_CIR_-X_OR_ encodes the amino acid changes T20A, A85T, T118I, and I127V. In addition, plasmids encoding PA-X_OR_ proteins containing single amino acid changes T20A, A85T, T118I, and I127V were transfected. At 24 h.p.t, Rluc expression (**B**,**C**) was quantified in a luminometer. Error bars represent the standard deviations from triplicates. *, *p* < 0.05; **, *p* < 0.005; ***, *p* < 0.0005; ****, *p* < 0.0001 using a Student’s *t*-test. R.u., relative units. (**D**) Protein expression levels of H5N1 PA-X and actin in HEK293T transfected cells were analyzed by Western blot using cells extracts and antibodies specific for the HA tag to detect PA-X proteins (upper blot) and actin (bottom blot). Molecular mass markers (in kDa) are indicated on the left. The experiments were repeated three times, with similar results.

The increased ability of H5N1 NS1_CIR_ to inhibit host gene expression and modulate innate immune responses is likely due to the amino acid changes L103F and I106M, as previously reported for H5N1 [24,25], H7N9 [55], and H1N1 [41] strains. However, less is known regarding the amino acids involved in the ability of H5N1 IAV PA-X protein to inhibit host gene expression. Therefore, to analyze which of the 8 amino acid changes different between PA-X_OR_ and PA-X_CIR_ were responsible for the increased ability of H5N1 PA-X_CIR_ to inhibit host gene expression, we introduced amino acid changes T20A, A85T, T118I, and I127V in PA-X_OR_ (named as PA_CIR_-X_OR_), and amino acid changes G209E, V212A, L221R, and R250Q in PA-X_CIR_ (named as PA_OR_-X_CIR_) (Figure 3A). Human HEK293T (Figure 3B) and avian DF-1 (Figure 3C) cells were transiently co-transfected with pCAGGS Rluc reporter expression plasmid together with pCAGGS plasmids encoding HA epitope-tagged H5N1 PA-X_OR_, PA-X_CIR_, PA_CIR_-X_OR_, and PA_OR_-X_CIR_. Then, at 24 h p.t, levels of Rluc (Figure 3B,C) expression were determined. Interestingly, levels of Rluc were similar in cells transfected with plasmids expressing H5N1 PA-X_OR_ and PA_OR_-X_CIR_. Likewise, levels of Rluc were similar in cells transfected with plasmids encoding H5N1 PA-X_CIR_ and PA_CIR_-X_OR_, which were lower than cells transfected with pCAGGS plasmids encoding H5N1 PA-X_OR_ and PA_OR_-X_CIR_ (Figure 3B,C). Correlating with these data, levels of H5N1 PA-X_OR_ and PA_OR_-X_CIR_ expression, as measured by Western blot with an antibody specific for the HA epitope tag, were similar, and higher than the levels of protein expression from H5N1 PA-X_CIR_ and PA_CIR_-X_OR_ transfected cells (Figure 3D). These data suggest that any or all of the amino acid changes (e.g., T20A, A85T, T118I, and I127V) are responsible for the higher ability of H5N1 PA-X_CIR_ to inhibit host gene expression, compared with H5N1 PA-X_OR_. To analyze the contribution of H5N1 PA-X amino acid changes T20A, A85T, T118I, and I127V on PA-X-mediated inhibition of host gene expression, amino acid changes were introduced individually in H5N1 PA-X_OR_. Then, human HEK293T (Figure 3B) and avian DF-1 (Figure 3C) cells were transiently co-transfected with the pCAGGS expression plasmid encoding Rluc together with pCAGGS plasmids encoding HA epitope-tagged H5N1 PA-X_OR_, PA-X_CIR_, and PA-X_OR_-T20A, PA-X_OR_-A85T, PA-X_OR_-T118I, and PA-X_OR_-V127I mutants, and levels of Rluc expression determined at 24 h p.t (Figure 3B,C). Levels of Rluc expression in cells expressing PA-X_OR_-T20A and PA-X_OR_-A85T were similar to those from cells transfected with PA-X_OR_ (Figure 3B,C). By contrast, levels of Rluc expression were reduced in cells expressing PA-X_OR_-T118I and PA-X_OR_-V127I mutants (Figure 3B,C). To further validate these results, PA-X protein expression levels were evaluated by Western blot (Figure 3D). Among the single mutants, PA-X_OR_-T118I was the one expressing the lowest levels (Figure 3D). Though mutants PA-X_OR_-T20A and PA-X_OR_-A85T did not efficiently inhibit host gene expression, PA-X_OR_-A85T was expressed at lower levels than PA-X_OR_-T20A. Although the reasons for these differences in protein expression levels between the different PA-X mutants were not directly addressed, it could be due to protein stability, as it has been shown that the half-life of PA-X ranges from approximately 30 min to 3.5 h depending on the IAV strain [51]. These data indicated that, among the different residues in H5N1 IAV PA-X, amino acid changes V127I, and particularly T118I, increase the ability of PA-X to inhibit host gene expression.

**Figure 4:**
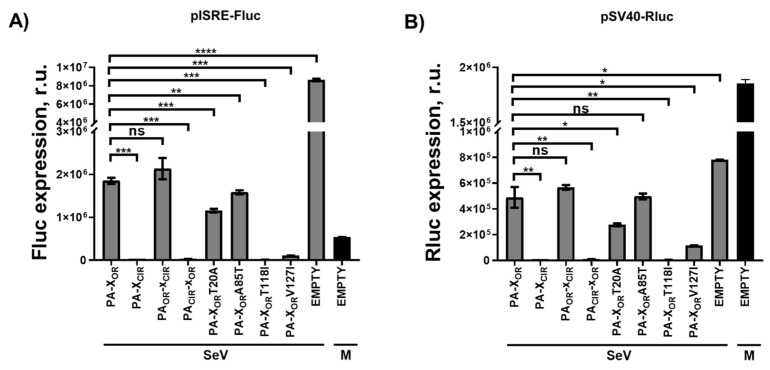
Effect of H5N1 NS1 and PA-X amino acid changes on IFN responses. (**A**,**B**) Human HEK293T cells were transiently co-transfected with a pCAGGS empty plasmid or with pCAGGS plasmids expressing the indicated HA epitope-tagged PA-X variants, together with a plasmid expressing Fluc under an ISRE dependent promoter, and a pCAGGS plasmid constitutively expressing Rluc. PA-X_CIR_ protein encodes the amino acid changes T20A, A85T, T118I, I127V, G209E, V212A, L221R, and R250Q. PA_OR_-X_CIR_ encodes the amino acid changes G209E, V212A, L221R, and R250Q. PA_CIR_-X_OR_ encodes the amino acid changes T20A, A85T, T118I, and I127V. At 24 h.p.t, cells were infected with SeV (Cantell strain, MOI 3) or left mock-infected (M), and 20 h later expression of Fluc (**A**) and Rluc (**B**) was analyzed by luminescence. Data show the mean and standard deviations from cells in triplicate. ns, non-significant; *, *p* < 0.05; **, *p* < 0.005; ***, *p* < 0.0005; ****, *p* < 0.0001 using a Student’s *t*-test. Experiments were repeated 3 times in triplicate, with similar results. R.u., relative units.

To further analyze if the ability to block host gene expression correlated with the ability of PA-X proteins to inhibit IFN responses during infection, HEK293T cells were co-transfected with pCAGGS plasmids expressing the different H5N1 PA-X variants together with the ISRE Fluc plasmid, and pCAGGS-Rluc (Figure 4). At 24 h p.t, cells were mock-infected or infected with SeV, Cantell strain, (MOI of 3), and Fluc (Figure 4A) and Rluc (Figure 4B) levels were evaluated at 24 h p.i. Interestingly, Fluc and Rluc expression data correlated with similar and higher Fluc and Rluc levels in PA-X_CIR_ and PA_CIR_-X_OR_ transfected cells, compared to PA-X_OR_ and PA_OR_-X_CIR_ transfected cells (Figure 4A,B). Moreover, amino acid change T118I, and, to a lower extent, V127I, increased the ability of H5N1 PA-X_OR_ to inhibit ISRE-dependent responses (Figure 4A), with increased ability to induce host shutoff (Figure 4B). These data suggested that amino acid changes T118I and I127V are responsible for the ability of H5N1 PA-X_CIR_ to inhibit host gene expression and that this shutoff activity is likely affecting IFN-dependent transcripts.

### 3.5. Effect of H5N1 NS1 and PA-X Mutations on Viral Growth

To analyze whether amino acid changes in H5N1 NS1 and PA-X modulating host shutoff and IFN responses affect viral replication, we generated four recombinant viruses: PA_OR_/NS1_OR_, PA_OR_/NS1_CIR_, PA_CIR_/NS1_OR_, and PA_CIR_/NS1_CIR_. Since IAV NS1 open reading frame (ORF) partially overlaps with the nuclear export protein (NEP), we used an NS segment encoding non-overlapping NS1 and NEP ORFs [36,42,43,44]. All the recombinant viruses encoded the PR8 segments with the exception of NS1, which were from H5N1 IAV circulating in 1996–1997 (NS1_OR_) or since 2016 (NS1_CIR_); and the H5N1 PA viral segment, which was from H5N1 viruses circulating during 1996–1997 (PA_OR_), or containing the PA-X mutations of H5N1 viruses circulating since 2016 (PA_CIR_). Consequently, for the PA segment, the PA-X_CIR_ protein encoded the amino acid changes T20A, A85T, T118I, I127V, G209E, V212A, L221R, and R250Q, and the PA_CIR_ encoded the amino acid substitutions T20A, A85T, T118I, and I127V, which affect both the PA and PA-X proteins.

**Figure 5:**
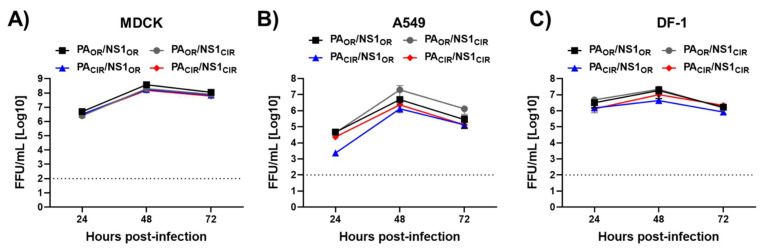
Growth kinetics of recombinant viruses containing amino acid changes in H5N1 IAV NS1 and PA proteins: MDCK (**A**), A549 (**B**), or DF-1 (**C**) cells were infected (MOI of 0.001 for MDCK or 0.01 for A549 and DF-1) in triplicates with the recombinant viruses encoding the indicated H5N1 NS1 and PA proteins (PA_OR_/NS1_OR_, PA_OR_/NS1_CIR_, PA_CIR_/NS1_OR_, and PA_CIR_/NS1_CIR_). Virus titers in cell culture supernatants were determined at the indicated h p.i. by immunofocus assay (FFU/mL). No statistically significant differences among PA_OR_/NS1_OR_ vs. PA_OR_/NS1_CIR_, PA_CIR_/NS1_OR_, or PA_CIR_/NS1_CIR_, using 2-way ANOVA (*p* < 0.001), were found.

To assess whether the mutations incorporated in the H5N1 NS1 and PA-X proteins affect virus growth, canine MDCK, human A549, or avian DF-1 cells were infected at low MOI (0.001 for MDCK and 0.01 for A549 and DF-1 cells), and virus titers in tissue culture supernatants were measured at 24, 48, and 72 h p.i. All viruses grew similarly in the three cell lines and no significant differences were observed (Figure 5), although viruses containing PA_OR_ (PA_OR_/NS1_OR_ and PA_OR_/NS1_CIR_) grew to slightly higher titers compared with viruses encoding the PA_CIR_ at 24 and 48 h p.i. (Figure 5).

### 3.6. Effect of H5N1 NS1 and PA-X Mutations on Viral Pathogenesis In Vivo

**Figure 6:**
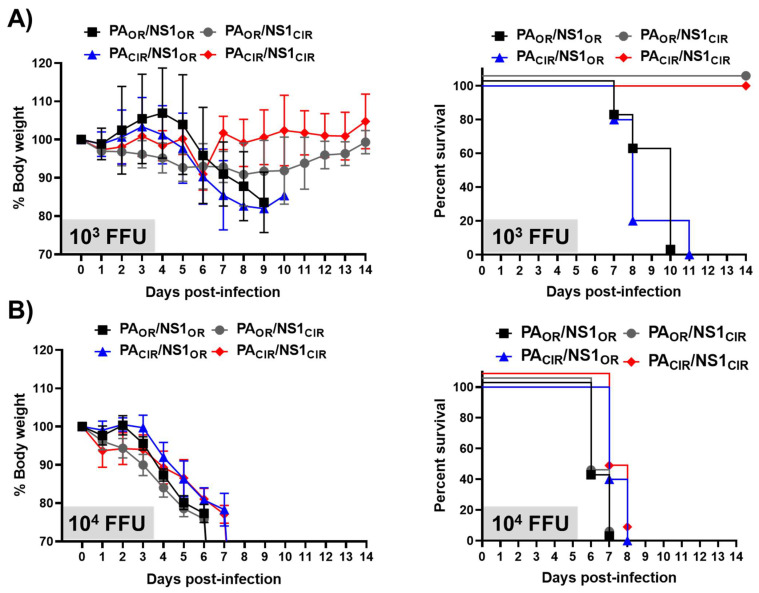
Virulence of recombinant viruses containing amino acid changes in H5N1 NS1 and PA proteins: Groups of 7-to-8-week-old A129 female mice (*N* = 5/group) were infected with 1000 (10^3^; **A**) or 10,000 (10^4^; **B**) FFU/mouse of the recombinant viruses encoding the indicated H5N1 NS1 and PA proteins (PA_OR_/NS1_OR_, PA_OR_/NS1_CIR_, PA_CIR_/NS1_OR_, PA_CIR_/NS1_CIR_). Weight loss (left panels) and survival (right panels) were evaluated daily for 2 weeks.

To analyze the effect of H5N1 NS1 and PA-X amino acid changes in viral pathogenesis and on the induction of innate immune responses, A129 mice were infected with 1000 and 10,000 FFU of the different viruses per mice. First, we evaluated weight loss and mortality during 14 days (Figure 6). Mice infected with 1000 FFU of the viruses encoding H5N1 NS1_CIR_ and either PA_OR_ or PA_CIR_ (PA_OR_/NS1_CIR_ and PA_CIR_/NS1_CIR_) did not significantly lose weight (Figure 6A), and none of them succumbed to viral infection (Figure 6B). However, mice infected with the viruses encoding H5N1 NS1_OR_ and either PA_OR_ or PA_CIR_ (PA_OR_/NS1_OR_ and PA_CIR_/NS1_OR_) started losing weight at day 6 p.i., and all of them succumbed to viral infection by days 10 or 11, respectively (Figure 6A,B, respectively). All the mice infected with 10,000 FFU, irrespective of the recombinant virus used in the infections, lost weight (Figure 6A) and all of them died from viral infection (Figure 6B). These data suggested that viruses encoding H5N1 NS1_OR_ (PA_OR_/NS1_OR_ and PA_CIR_/NS1_OR_) were more virulent than viruses encoding H5N1 NS1_CIR_ (PA_OR_/NS1_CIR_ and PA_CIR_/NS1_CIR_) and that the presence of H5N1 PA_OR_ or PA_CIR_ did not significantly affect viral pathogenesis, at least in the A129 mouse model of infection, and with experimental conditions used in these studies.

**Figure 7:**
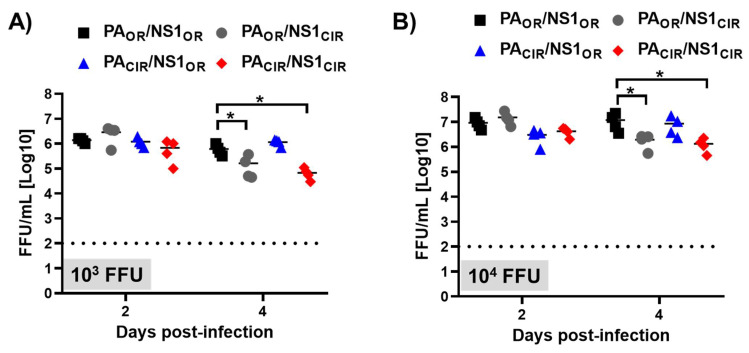
Viral titers of recombinant viruses containing amino acid changes in H5N1 NS1 and PA proteins: Groups of 7-to-8-week-old A129 male mice (*N* = 8/group) were infected with 1000 (10^3^; **A**) or 10,000 (10^4^; **B**) FFU/mouse of the indicated viruses (PA_OR_/NS1_OR_, PA_OR_/NS1_CIR_, PA_CIR_/NS1_OR_, and PA_CIR_/NS1_CIR_). Mice were sacrificed at 2 (*N* = 4) and 4 (*N* = 4) d p.i, and right lungs were harvested, homogenized, and used to quantify viral titers by immunofocus assay (FFU/mL. *, *p* < 0.05 (PA_OR_/NS1_OR_ vs. PA_OR_/NS1_CIR_, PA_CIR_/NS1_OR_, and PA_CIR_/NS1_CIR_) using 2-way ANOVA (*N* = 4 per time point). Non-significant when *p* > 0.05.

To study whether the higher virulence of viruses encoding H5N1 NS1_OR_ (PA_OR_/NS1_OR_ and PA_CIR_/NS1_OR_) correlated with higher virus titers in the lungs of infected animals, another set of A129 mice was similarly infected with 1000 and 10,000 FFU/mice, and viral titers were analyzed at days 2 and 4 after infection (Figure 7). Viral titers in the lungs of animals inoculated with 1000 or 10,000 FFU were similar for the four viruses at day 2 p.i. Interestingly, viral titers at day 4 p.i were slightly higher (between 4- to 8-fold) for viruses encoding H5N1 NS1_OR_ (PA_OR_/NS1_OR_ and PA_CIR_/NS1_OR_) than viruses encoding H5N1 NS1_CIR_ (PA_OR_/NS1_CIR_ and PA_CIR_/NS1_CIR_) for both viral doses (Figure 7A,B). These data correlate with the higher virulence observed for H5N1 NS1_OR_-encoding viruses (PA_OR_/NS1_OR_ and PA_CIR_/NS1_OR_) (Figure 6)_._

**Figure 8:**
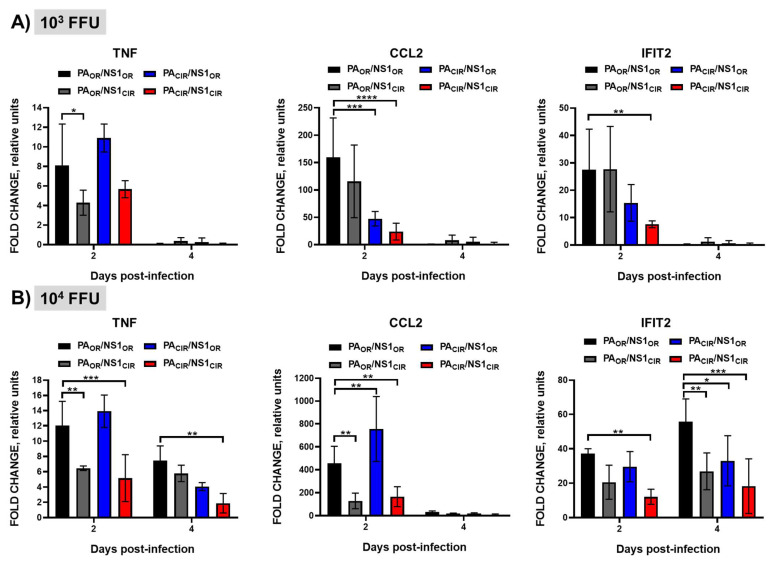
Induction of innate immune responses in vivo by recombinant viruses encoding H5N1 NS1 and/or PA variants: Groups of 7-to-8-week-old A129 male mice (*N* = 8/group) were infected with 1000 (10^3^; **A**) or 10,000 (10^4^; **B**) FFU/mouse of the indicated viruses (PA_OR_/NS1_OR_, PA_OR_/NS1_CIR_, PA_CIR_/NS1_OR_, and PA_CIR_/NS1_CIR_) or left mock-infected (*N* = 4). Mice were sacrificed at 2 (*N* = 4) and 4 (*N* = 4) d p.i. Left lungs were collected and total RNA was extracted to quantify the levels of TNF (left panels), CCL2 (middle panels), and IFIT2 (right panels) by RT-qPCR. Levels of expression in infected mice were compared to those in mock-infected mice. *, *p* < 0.05; **, *p* < 0.005; ***, *p* < 0.0005; ****, *p* < 0.0001 (PA_OR_/NS1_OR_ vs. PA_OR_/NS1_CIR_, PA_CIR_/NS1_OR_, and PA_CIR_/NS1_CIR_) using 2 way ANOVA (*n* = 4 per time point). Non-significant when *p* > 0.05.

It has been shown that increased levels of pro-inflammatory cytokine induction correlate with higher virulence of H5N1 IAV [11]. To analyze whether the higher virulence of H5N1 NS1_OR_-encoding viruses (PA_OR_/NS1_OR_ and PA_CIR_/NS1_OR_) correlated with higher induction of pro-inflammatory cytokines (TNF, CCL2) or interferon-stimulated genes (ISGs, IFIT2), and whether the induction of these genes correlated with the shutoff activity of NS1 and PA-X, mRNA levels in the lungs of mice infected with 1000 and 10,000 FFU/mice were measured by RT-qPCR at 2 and 4 d p.i (Figure 8A,B). Expression of TNF, CCL2, and IFIT2 was induced in the lungs of infected compared to mock-infected control lungs, with the exception of mice infected with 1000 FFU at 4 d p.i. Interestingly, at 2 d p.i, expression of these cytokines was higher in the lungs of A129 mice infected with the highest dose of viruses encoding H5N1 NS1_OR_, than those mice infected with H5N1 NS1_CIR_-encoding viruses (Figure 8A,B). Moreover, expression of TNF and IFIT2 at 4 d p.i in mice infected with 10,000 FFU and expression of CCL2 and IFIT2 at 2 d p.i in mice infected with 1000 FFU, were higher in A129 mice infected with H5N1 PA_OR_ and NS1_OR_ (PA_OR_/NS1_OR_) and lower in A129 mice infected with H5N1 PA_CIR_ and NS1_CIR_ (PA_CIR_/NS1_CIR_) viruses. These data suggest that H5N1 NS1_CIR_, and, to a lower extent, PA-X_CIR_, contribute to decrease inflammatory responses after virus infection, likely due to the increased inhibition of host gene expression mediated by H5N1 NS1_CIR_ and PA-X_CIR_ compared with NS1_OR_ and PA-X_OR_, respectively.

## 4. Discussion

IAV evolve in their hosts to optimize fitness and transmission. In addition, to successfully replicate in their natural hosts and to evade antiviral responses induced during viral infection, IAV encode specific proteins, such as the NS1 and PA-X, to counteract host antiviral responses, allowing the virus to replicate in IFN competent systems [12]. In this work, we assessed the contribution of NS1 and PA-X proteins from different H5N1 IAV, as well as the role of specific amino acid mutations in the PA-X protein of H5N1 IAV on their ability to inhibit host gene expression, viral replication, and pathogenesis.

Influenza NS1 and PA-X subvert antiviral responses through the inhibition of host gene expression, including IFNs and ISGs, many of which display antiviral activity [12]. For IAV NS1 protein, one of the mechanisms to inhibit host gene expression involves its binding to CPSF30 [49,57]. This binding inhibits cellular pre-mRNA processing, causing a global inhibition of host gene expression [49,57]. However, there are other mechanisms by which IAV NS1 protein inhibits antiviral responses [12,13]. In fact, the property of inhibiting host gene expression is not conserved among all IAV strains [12]. Previously, we described that, whereas the NS1 protein from pH1N1 IAV circulating in 2009 did not inhibit host gene expression, 6 amino acid changes were selected in the viruses circulating afterward, leading to an NS1 protein able to induce cellular shutoff [36,42]. Similarly, in this work, we describe that whereas the NS1 protein from H5N1 IAV strains circulating during 1996–1997 do not efficiently inhibit host gene expression, likely including IFN responses, H5N1 viruses circulating afterward have selected amino acid changes in NS1 leading to an increase ability to induce cellular shutoff (Figure 1 and Figure 2). In accordance with our data, a previous study showed that amino acid changes L103F and I106M, which are also different between H5N1 NS1_OR_ and NS1_CIR_, increase the ability of H5N1 NS1 to inhibit host gene expression and IFN responses [24,25] because these amino acid changes allow binding to CPSF30 [24]. Similarly, for influenza H7N9 strains, it has been shown that the NS1 amino acid change I106M can restore CPSF30 binding together with the ability to block host gene expression [55]. In the case of influenza H9N2 strains, amino acid substitutions L103F, I106M, P114S, G125D, and N139D in A/quail/Hong Kong/G1/97 H9N2 NS1 resulted in binding to CPSF30 and, in consequence, inhibition of host gene expression [58]. For avian H9N2 viruses, whereas the NS1 protein from recent isolates does not inhibit host gene expression, the NS1 proteins from earlier H9N2 strains does, suggesting that in the case of avian host infected with H9N2 strains, encoding a NS1 protein lacking the ability to inhibit host gene expression is beneficial for the virus [58].

We also showed that H5N1 IAV circulating nowadays encode a PA-X protein with eight amino acid changes (T20A, A85T, T118I, I127V, G209E, V212A, L221R, and R250Q) different from the PA-X proteins from H5N1 viruses circulating during 1996–1997, and that H5N1 PA-X_CIR_ displays increased ability to inhibit host gene expression as compared with PA-X_OR_ (Figure 1 and Figure 2). However, less is known about the amino acid residues in H5N1 PA-X contributing to its ability to inhibit host gene expression. We show that, among the eight amino acid differences between H5N1 PA-X_OR_ and PA-X_CIR_, two of them, T118I and V127I, are responsible for the increased ability of H5N1 PA-X to inhibit host gene expression, whereas amino acid changes T20A and A85T have little or no effect on PA-X´s host shutoff activity (Figure 3 and Figure 4). Notably, substitution I118T is located within the endonuclease active site in the PA-X N-terminal domain [59]. Similar to our results, using an H9N2 strain, it has been shown that amino acid change T118I increases the ability of PA-X to inhibit host gene expression, whereas the amino acid change T20A has no effect on PA-X-mediated inhibition of host gene expression [59]. Interestingly, four of these amino acid changes (T20A, A85T, T118I, and I127V), especially two of them that mostly affect PA-X´s activity (T118I, I127V), also affect the protein sequence of PA; therefore, it is difficult to know whether these identified amino acid changes were selected due to a beneficial effect on PA-X, PA, or both. Furthermore, the selection of these mutations could be influenced by amino acid changes that occurred somewhere else in the viral genome, including NS1 [36]. Notably, by analyzing the evolution of pH1N1 in humans, we found that there are multiple amino acid changes in NS1 and PA-X proteins from circulating seasonal viruses compared to the viruses isolated at the origin of the 2009 pandemic. These amino acid substitutions are responsible for increased NS1-mediated inhibition of host gene expression and decreased PA-X-mediated shutoff, including innate immune response genes [36]. Notably, a recombinant pH1N1 virus containing PA-X and NS1 genes from recently circulating pH1N1 viruses has better viral fitness and is more pathogenic compared with the original pH1N1 [36]. Recombinant viruses encoding the PA-X amino acid change R195K, showed decreased shutoff activity and showed increased replication and transmission in ferrets but not in chickens [60], suggesting that this amino acid change might have contributed to cross-species transmission of H7N9, H5N6, and H1N1/2009 viruses from animal reservoirs to humans [60]. In the case of canine IAV, genetic analysis indicated that within the PA-X, X-ORFs of equine-derived H3N8 and avian-derived H3N2 viruses encoded 61 amino acids but were truncated after introduction into dogs [61]. Truncation of PA-X suppressed expression from co-transfected plasmids in cells and enhanced viral pathogenicity and transmission in dogs, suggesting that truncation of PA-X might be important for the adaptation of influenza viruses to dogs [61]. Importantly, these results demonstrate an evolution in the abilities of NS1 and PA-X proteins to induce host shutoff and show how this is beneficial for IAV. In addition, several in vitro studies have shown that multiple amino acids in PA-X can affect PA-X´s shutoff activity, and that differences between IAV strains or subtypes can occur [36,38,39,56,62,63].

To analyze the combined effect of H5N1 NS1 and PA-X amino acid changes on virus replication, pathogenesis, and the induction of innate immune responses, we generated four recombinant viruses (PA_OR_/NS1_OR_, PA_OR_/NS1_CIR_, PA_CIR_/NS1_OR_, and PA_CIR_/NS1_CIR_). All the recombinant viruses encoded the PR8 genes with the exception of NS1 and PA/PA-X ORFs, which were from H5N1 IAV circulating in 1996–1997 (NS1_OR_ and PA_OR_, respectively) or since 2016 (NS1_CIR_ and PA_CIR_, respectively). Consequently, for the PA segment, the PA-X_CIR_ protein encoded the amino acid changes T20A, A85T, T118I, I127V, G209E, V212A, L221R, and R250Q, and the PA_CIR_ encoded the amino acid substitutions T20A, A85T, T118I, and I127V, which affect both to the PA and PA-X proteins.

Remarkably, viruses encoding the NS1_OR_ were more virulent in A129 mice, measured by body weight losses and survival rates (Figure 6), and replicated slightly more efficiently in the lungs of infected A129 mice than the viruses encoding H5N1 NS1_CIR_ (Figure 7). To analyze possible reasons for these changes in virulence, we analyzed the response of pro-inflammatory cytokines and ISGs after viral infection. Inflammatory response has two distinct roles in the pathogenesis of IAV. Whereas activation is protective against viral infection, an uncontrolled response can cause severe damage. Interestingly, viruses encoding H5N1 NS1_OR_ (PA_OR_/NS1_OR_ and PA_OR_/NS1_CIR_) induced increased levels of TNF, CCL2, and IFIT2 expression (Figure 8A,B) compared with viruses encoding H5N1 NS1_CIR_ (PA_OR_/NS1_CIR_ and PA_CIR_/NS1_CIR_). This is likely due to the reduced ability of H5N1 NS1_OR_ to mediate cellular shutoff compared with H5N1 NS1_CIR_ (Figure 2). These highest levels of pro-inflammatory cytokine induction observed for viruses encoding H5N1 NS1_OR_ are likely responsible for their increased virulence (Figure 6 and Figure 7). In fact, it has been shown that increased levels of pro-inflammatory cytokines and viral loads correlate with higher virulence with H5N1 IAV [11]. Similarly, mice and macaques infected with seasonal H1N1 virus induced lower levels of pro-inflammatory cytokines and chemokines than animals infected with the 2009 pH1N1 virus, resulting in decreased pathogenicity [64]. In pigs, two swine-origin pH1N1 viruses, derived from a human patient and from swine, were more virulent than a swine-origin 1918-like classical IAV [65]. Interestingly, these IAV induced higher expression of pro-inflammatory genes compared with the swine-origin 1918-like classical IAV, suggesting that both pH1N1 isolates are more virulent, at least in part, due to differences in the host transcriptional response during acute infection [65].

Altogether, our data suggest that circulating H5N1 IAV has adapted to induce less inflammatory responses and be less virulent than original circulating H5N1 IAV. However, additional experiments are needed to further analyze these differences. We are aware of the limitations from our study because it will be important to test this hypothesis in different animal models, including avian hosts. Moreover, recombinant viruses containing the original and modified PA/PA-X and/or NS1 proteins in the backbone of an H5N1 IAV should be used. Finally, because NS1 and PA/PA-X are multifunctional proteins, the specific role of the amino acid changes observed between original and circulating strains needs further analysis. In any case, our results indicate a functional co-evolution of PA-X and NS1 in H5N1 IAV, which is important to inhibit host gene expression, as we have shown previously [36,37]. Remarkably, our results also demonstrate the importance of conducting H5N1 IAV surveillance and to monitor these and other mutations contributing to H5N1 IAV pathogenesis to predict and hopefully prevent potential future H5N1 IAV pandemics.

## Data Availability

The data that support the findings of this study are available from the corresponding author upon reasonable request.
